# NMDA receptors are not necessary for burst firing of lateral habenula neurons in mice

**DOI:** 10.3389/fpsyt.2026.1828920

**Published:** 2026-06-11

**Authors:** Rong Zhou, Jingjing Lu, Yunxiang Ling, Hong Wang, Lijun Cui, Yongliang Pan, Huanxin Chen

**Affiliations:** 1 Key Lab of Psychiatry Medicine, Research Institute of Psychiatry Medicine, Huzhou Third Municipal Hospital, the Affiliated Hospital of Huzhou Normal University, Huzhou, Zhejiang, China; 2 School of Medicine, Huzhou Normal University, Huzhou, Zhejiang, China

**Keywords:** burst firing, D-AP5, MK-801, NMDA receptor, the lateral habenula, T-type calcium channel

## Abstract

**Background:**

Burst firing in the lateral habenula (LHb) is enhanced under stress and depression, and ketamine blocks burst firing in LHb to exert a rapid antidepressant effect. Thus, burst firing in LHb may serve as a cellular mechanism of depression and a promising therapeutic target for developing novel antidepressants. However, the mechanisms underlying burst firing in LHb are not fully understood. The present study aims to investigate the contribution of N-methyl-D-aspartate receptor (NMDAR) to burst firing in LHb.

**Methods:**

C57BL/6J male mice aged 8 to 10 weeks were used in the present study. Whole-cell recording was performed in brain slices containing LHb. Spontaneous and rebound burst firings were recorded in current-clamp mode. The effect of NMDAR antagonists, D-2-Amino-5-phosphonovaleric acid (D-AP5) and dizocilpine (MK-801), on burst firing was assessed.

**Results:**

Spontaneous burst firing in LHb neurons persisted in the presence of a specific NMDAR antagonist, D-AP5. The percentages of neurons with spontaneous burst firing, burst firing frequency, and spike number per burst were not different between in D-AP5-containing bath solution and in control bath solution. D-AP5 also did not affect ongoing spontaneous burst firing. Rebound burst firing was not affected in the presence of D-AP5, nor was ongoing rebound burst firing. Likewise, MK-801, a use-dependent, non-competitive NMDAR antagonist, did not affect rebound burst firing.

**Conclusions:**

NMDARs are not necessary for the generation of burst firing in LHb neurons, although they may play a modulating role under certain states.

## 1 Introduction

The lateral habenula (LHb) serves as a critical integrator between the forebrain and midbrain. A major output of LHb targets and negatively regulates midbrain monoaminergic neurons, including dopaminergic and serotonergic populations (1, 2). Notably, the activity in LHb dramatically increases under aversive and stressful states, and thus, LHb has been recognized as “an anti-reward center” in the brain (3). Consistently, hyperactivity in LHb has been observed in patients with major depressive disorder (MDD) and in animal models of depression (4–6). Furthermore, direct stimulation of LHb in MDD patients elicits a rapid antidepressant effect (7–9). Collectively, these findings underscore the pivotal role of LHb in the pathophysiology of depression.

LHb neurons are predominantly glutamatergic and display dynamic firing properties (5, 10). In rodent models of depression, the percentage of LHb neurons exhibiting spontaneous burst firing is markedly elevated. Moreover, rebound burst firing induced by hyperpolarizing pulses in naive animals has also been shown to trigger depressive-like behaviors (5). It was also found that ketamine exerts its rapid antidepressant effects by suppressing burst firing in LHb neurons (5, 11, 12). Together, these findings identify burst firing in LHb neurons as a key neural substrate underlying depression and a promising therapeutic target (13). However, the cellular mechanisms underlying the burst firing remain incompletely understood.

Burst firing is a common neuronal firing mode observed across diverse brain regions, including the hippocampus, thalamus, and midbrain dopaminergic nuclei (14–19). While T-type calcium channels are essential for the generation of burst firing in many neuronal types, N-methyl-D-aspartate receptors (NMDARs) have also been implicated in some brain regions (20–23). Recent studies have reported that burst firing in LHb neurons requires concurrent activation of T-type calcium channels and NMDARs, and that NMDAR antagonists abolish both spontaneous and rebound burst firing in LHb neurons (2, 5, 13).

In contrast to previous reports (5, 6), we found that burst firing in LHb neurons persisted when NMDARs were blocked. In the present study, we used whole-cell recordings in the brain slices containing LHb to investigate the role of NMDARs in regulating both spontaneous and rebound burst firing. We observed that neither form of burst firing was blocked by the selective NMDAR antagonist D- (–)-2-Amino-5-phosphonovaleric acid (D-AP5). Likewise, dizocilpine (MK-801), a use-dependent, non-competitive NMDAR antagonist analogous to ketamine, did not alter rebound burst firing. These results differ from previous reports (5, 6), and suggest that NMDARs may not be necessary for the initiation of burst firing in LHb neurons. Future studies are warranted to clarify the contribution of NMDARs to burst firing in LHb neurons under physiological and pathological conditions.

## 2 Materials and methods

### 2.1 Animals

C57BL/6J male mice aged 8 to 10 weeks were used in the present study. The mice were purchased from GemPharmatech (Jiangsu, China) and housed under standard laboratory lighting conditions (light on between 8:00 and 20:00) and temperature (22 ± 1 °C) with food and water ad libitum. All experiment procedures were approved by the Ethics Committee on Animal Care and Use of Huzhou Third Municipal Hospital (Approval No. 202112017) and performed in accordance with the NIH Guide for the Care and Use of Laboratory Animals.

### 2.2 Brain slice preparation

Mice were anesthetized with isoflurane inhalation and perfused transcardially with ice-cold sucrose-based cutting solution (in mM): 210 Sucrose, 26 NaHCO_3_, 1.25 NaH_2_PO_4_, 2.5 KCl, 1 CaCl_2_, 6 MgCl_2_, 20 D-glucose. After decapitation, whole brains were rapidly removed. Coronal brain slices (300 to 350 µm thick) containing LHb were cut using a vibrating microtome (VT1200s, Leica Microsystems) in the ice-cold cutting solution gassed with 95% O_2_/5% CO_2_ to a pH of 7.4. The slices were quickly transferred to an incubator with pre-warmed artificial cerebrospinal fluid (ACSF in mM: 124 NaCl, 26 NaHCO_3_, 1.25 NaH_2_PO_4_, 2.5 KCl, 2 CaCl_2_, 2 MgCl_2_, and 10 D-glucose gassed with 95% O_2_/5% CO_2_) at 36 °C. After 45 min of warm incubation, the slices were maintained at room temperature (22 ± 0.5 °C). For electrophysiological recording, individual slices were transferred to a submerged recording chamber after at least 1 hour of incubation at room temperature and perfused continuously (1.5 mL/min) with oxygenated ACSF with 2 mM CaCl_2_ and 1 mM MgCl_2_. The temperature in the recording chamber was maintained at 29 to 30 °C (TC-344C, Warner Instruments).

### 2.3 Electrophysiological recordings

Whole-cell recordings were made on LHb neurons using the Multiclamp 700B amplifier (Molecular Devices, San Jose, CA). Neurons were visualized and recorded under infrared differential interference contrast (IR-DIC) microscopy with a fixed stage microscope (Axioskop-FS; Olympus BX51WI, Japan) equipped with a 40X water-immersion objective and a digital camera (C11440, Hamamatsu, Japan). Borosilicate glass pipettes were pulled using a P-1000 pipette puller (Sutter Instrument, USA), with resistances ranging from 3–6 MΩ when filled with internal solution. The internal solution used for recording spontaneous and rebound burst firing contained (in mM): 125 K-gluconate, 8 NaCl, 10 HEPES, 4 MgATP, 0.3 Na_3_GTP, 0.2 EGTA, 10 phosphocreatine, 0.1% biocytin (pH 7.2 adjusted with KOH, osmolarity 290–300 mOsM). To record NMDA components of excitatory postsynaptic currents (EPSCs), K-gluconate was replaced by Cs-methanesulfonate in the internal solution. Signals were acquired with pClamp 11 software (Molecular Devices), digitized at 10 kHz, and filtered at 2 kHz. Offline analysis was performed with Clampfit 10 (Molecular Devices).

Whole-cell recording was accepted for analysis when it remained stable after the rupture with an access resistance ≤ 25 MΩ. Resting membrane potential (RMP) was measured immediately after membrane rupture in current-clamp mode (I = 0). The intrinsic properties of neurons were determined by delivering a series of currents (from -100 to 200 pA, 500ms duration, 20 pA increment). Then, neurons were recorded continuously for at least 2 minutes in current-clamp mode at RMP to determine their category as silent, tonic firing, or burst firing neurons. Silent or tonic firing neurons were then held at -60 to -75 mV to determine whether they changed to burst firing mode. A burst firing was defined as consisting of 3 or more action potentials riding on a calcium spike. Neurons with spontaneous burst firing were continuously recorded for at least 5 min. Rebound burst firing was evoked by hyperpolarizing current pulses while holding neurons at -60 to -75 mV. To facilitate spontaneous and rebound burst firing of LHb neurons, mice underwent short-term restraint stress (2 h daily; 9:00 to 11:00) for 7 consecutive days, confined in ventilated 50 ml conical tubes. To record the NMDA component of evoked EPSC, neurons were voltage-clamped at +45 mV. Electrical stimulation was delivered by an ACSF-filled glass pipette positioned near the perisomatic region. 2,3-dioxo-6-nitro-7-sulfamoyl-benzo quinoxaline (NBQX) was included in the bath solution to block α-amino-3-hydroxy-5-methyl-4-isoxazolepropionic acid (AMPA) components, and picrotoxin to block gamma-aminobutyric acid (GABA) components.

The effects of NMDAR antagonists (D-AP5 and MK-801) on spontaneous and rebound burst firing were examined using two experimental approaches. In one approach, recordings were obtained in the continuous presence of NMDAR antagonists in bath solution. In the other, baseline recordings were first collected under control conditions, followed by the subsequent application of NMDAR antagonists.

### 2.4 Data statistical analysis

Data were analyzed using GraphPad Prism 8.0 software (GraphPad Software, La Jolla, CA). For both spontaneous and rebound burst firing, the percentages of neurons exhibiting spontaneous burst firing or rebound burst firing, spontaneous burst firing frequency, and spike number per burst were collected. Comparisons were conducted between control and drug conditions, as well as before and after drug application within the same neurons. Given that up to 3 min was required for D-AP5 to completely block NMDAR component under our experiment condition, statistical comparisons of spontaneous burst firing(the frequency and spike number per burst) were based on 2 to 5 min recordings before and 3 min after drug application. The rebound burst firing (spike number per burst) was compared based on the average of 3 hyperpolarizing pulses at 0.05Hz before and at least 5 min after application of D-AP5 or MK-801. Data were expressed as mean ± SEM. For comparison, the Kolmogorov-Smirnov (K-S) test was used to test data normality, and the F test to assess variance homogeneity. An unpaired t-test was used for different groups, and a paired t-test was used to compare before and after drug administration for normally distributed data. The K-S test was used to compare data that are not in the normal distribution. Chi-square test was used for comparison of the percentages of neurons between different groups. Repeated-measure ANOVA was used for the effect of mibefradil at different time points, followed by Tukey’s multiple comparisons test. The statistical significance is defined as p < 0.05.

### 2.5 Chemicals

The chemicals used in this study include D-AP5, NBQX, picrotoxin, MK-801, and mibefradil. All were purchased from MCE (Shanghai). All drugs were first dissolved in distilled water as stock solutions and diluted to the desired concentrations in bath solution.

## 3 Results

### 3.1 Firing characteristics of LHb neurons

**Figure 1:**
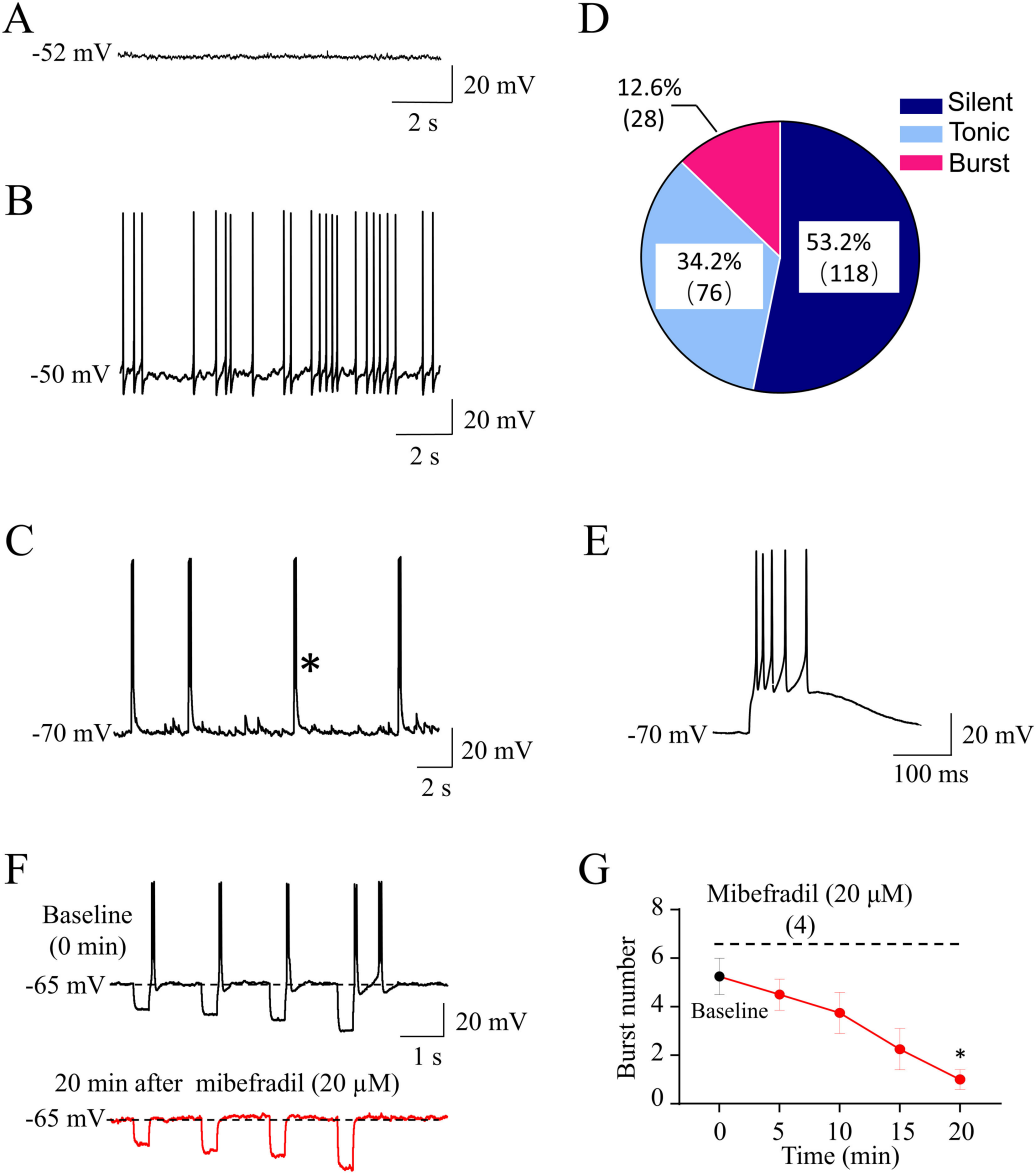
Firing characteristics of LHb neurons. **(A, C)** Representative traces from silent **(A)**, tonic **(B)**, and burst **(C)** neurons recorded at current-clamp mode. **(D)** A pie chart shows the percentage of each group; the number of neurons recorded is indicated in parentheses. **(E)** An expanded burst firing taken from C marked by a star. **(F)** Rebound burst firings induced by hyperpolarizing pulses of different intensities (20 pA, 40pA, 60 pA and 80 pA) before (upper black trace) and 20 min after application of mibefradil (20 μM), a T-type calcium channel antagonist (lower red trace). **(G)** Summary data show the effect of mibefradil on burst number at different times. The star indicates the significant difference between baseline and at 20 min after drug application (p = 0.01).

We initially performed recordings from naïve mice but observed a low incidence of spontaneous burst firing (6%, 3/50 neurons). The percentage of neurons exhibiting spontaneous burst firing increased to 12.6% (28/222 neurons) following short-term restraint stress. Consistent with previous reports (5), LHb neurons could be categorized into three firing types: silent (53.2%), tonic firing (34.2%), and burst firing (12.6%, **Figures 1A–E**). In our study, burst firing neurons included those exhibiting spontaneous burst firing at RMP or when held at -60 mV to -75 mV.

We tested if burst firing was dependent on T-type calcium channels with rebound burst firing. We found that specific T-type calcium channel antagonists completely abolished burst firing and calcium spikes. In those experiments, rebound burst firings were induced by current trains at 0.05Hz, each containing 4 hyperpolarizing pulses (350 ms) with increasing intensities (20 pA, 40 pA, 60 pA and 80 pA, **Figures 1F, G**). The number of rebound burst firing per train was gradually reduced after application of 20 μM mibefradil. It was 5.25 ± 0.75 at baseline (before drug application) and 1.0 ± 0.41 after 20 min (n = 4, p = 0.006, repeated measure ANOVA test; baseline vs 20 min, p = 0.01, Tukey’s test).

### 3.2 Spontaneous burst firing in LHb neurons persisted in the presence of NMDAR antagonists

**Figure 2:**
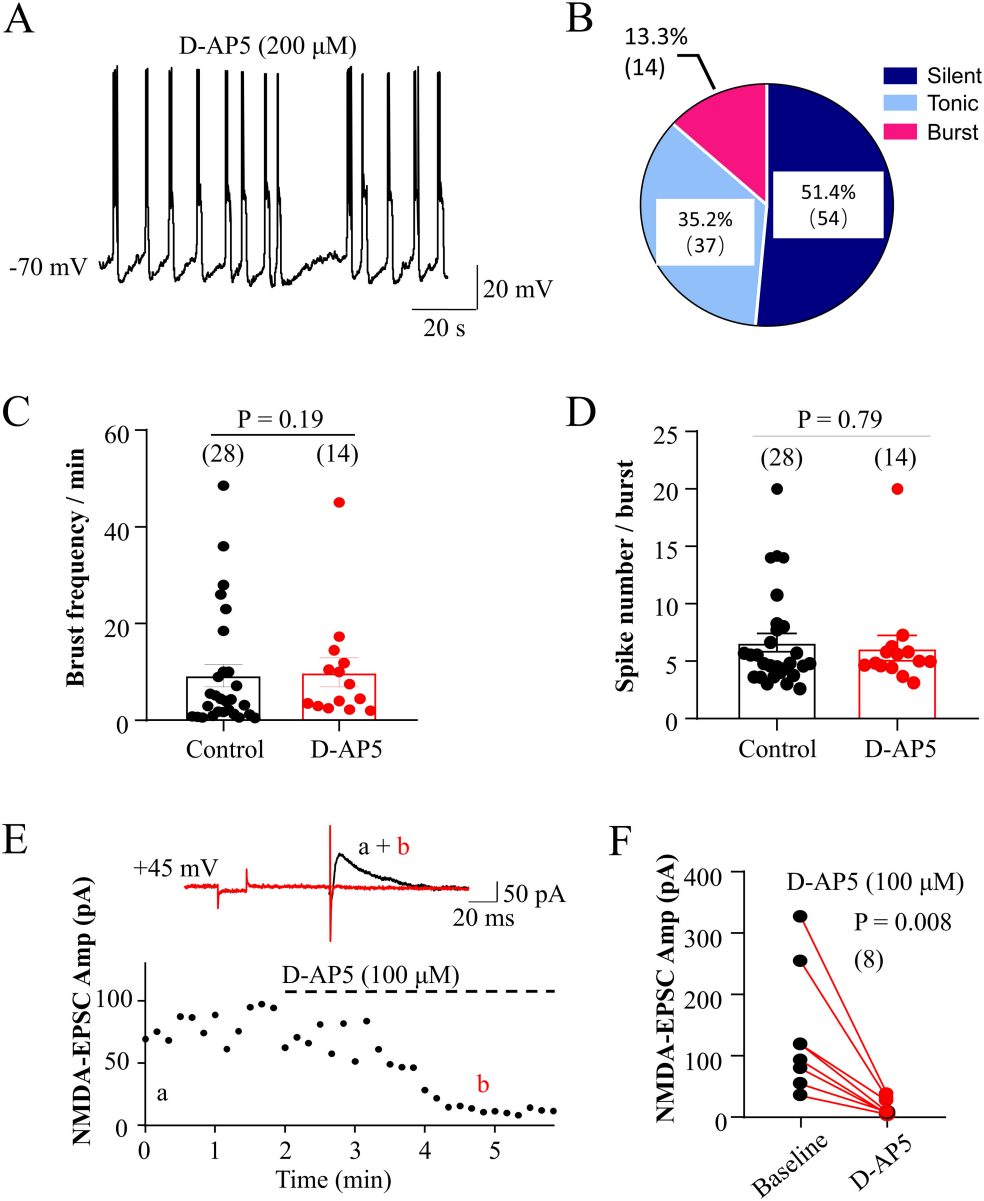
Spontaneous burst firing in LHb neurons persists in the presence of NMDAR antagonist D-AP5. **(A)** A representative spontaneous burst firing trace from an LHb neuron recorded in the presence of 200 µM D-AP5 in bath solution. **(B)** A pie chart shows the percentage of each group recorded in the presence of D-AP5. **(C, D)** Summarized data show spontaneous burst firing frequency**(C)** and spike number per burst **(D)** of LHb neurons recorded in the presence (red) and absence (black) of D-AP5. **(E)** A representative experiment shows the effect of D-AP5 (100 μM) on NMDA component of evoked EPSC. Insert traces show NMDA components before (black) and 3 min after (red) application of D-AP5. Letters indicate where traces are taken. **(F)** Summarized data show the effect of D-AP5 on the amplitude of NMDA components (baseline, black; 3 min after application of D-AP5, red).

Previous studies reported that spontaneous burst firing in LHb neurons is abolished by specific NMDAR antagonist D-AP5 and ketamine (5, 6, 24), indicating an essential requirement for NMDARs. In contrast, we still obtained neurons with spontaneous burst firing in brain slices which had been perfused with D-AP5 in different concentrations (100 μM, n = 8; 200 μM, n = 6; **Figure 2A**). We found that the percentage of neurons with spontaneous burst firing was not different between control and D-AP5-treated groups (control: 28/222 neurons; D-AP5: 14/105 neurons; p = 0.40, Chi-square test; **Figure 2B**). No significant changes were found in spontaneous burst firing frequency (control: 9.23 ± 2.32 per min, n = 28; D-AP5 group: 9.89 ± 3.01 per min, n = 14; p = 0.19, K-S test) or spike number per burst (control: 6.63 ± 0.80; D-AP5 group: 6.14 ± 1.10; p = 0.79, K-S test) (**Figures 2C, D**). To verify effective NMDAR blockade, we confirmed that 100 µM D-AP5 significantly eliminated the NMDA component of evoked EPSC within 3 min (baseline: 134.4 ± 36.01 pA; 3 min after D-AP5 application: 15.72 ± 4.60 pA; n = 8, p = 0.008, paired t-test; **Figures 2E, F**).

**Figure 3:**
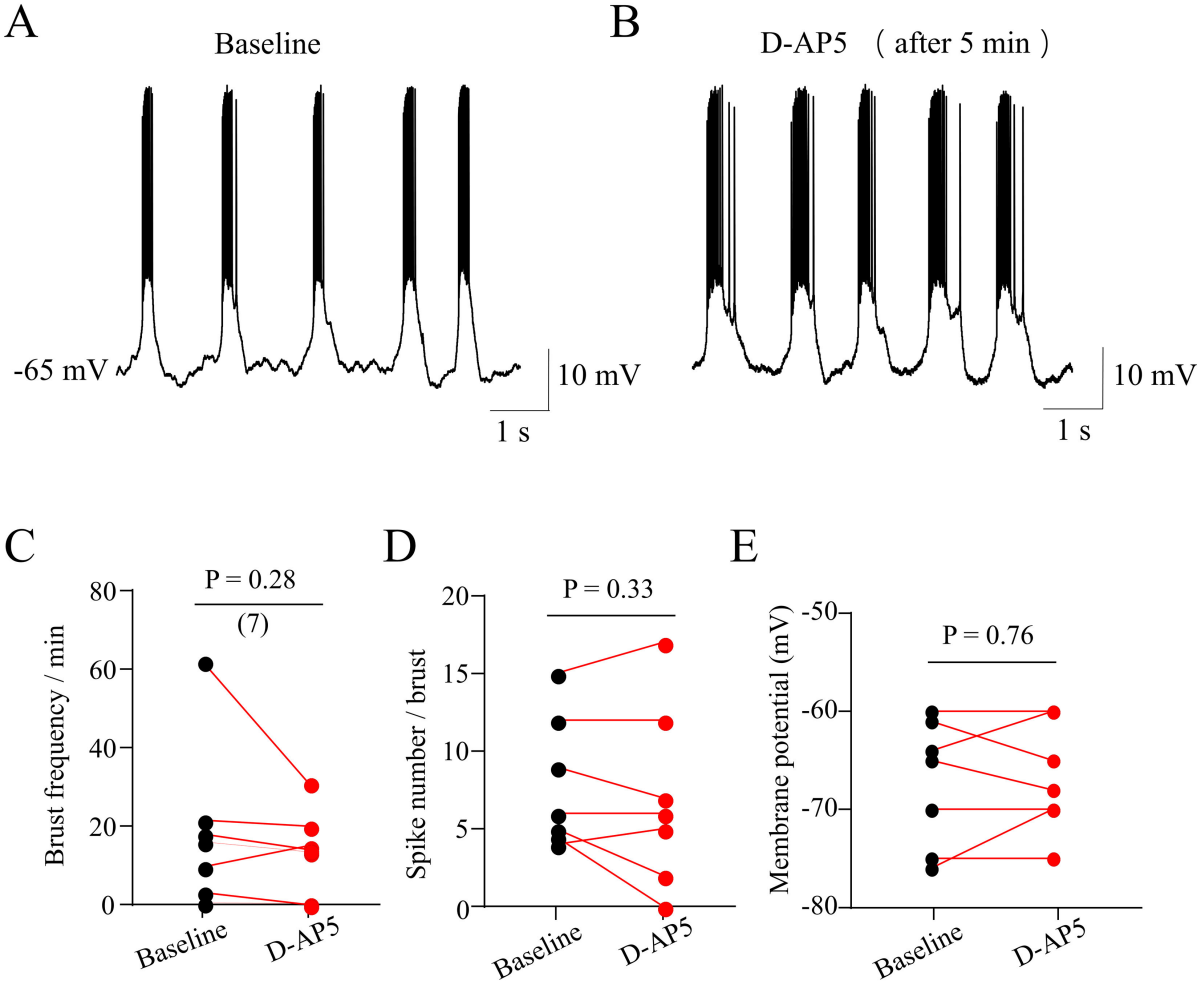
D-AP5 does not suppress ongoing spontaneous burst firing. **(A, B)** A representative spontaneous burst firing trace from a neuron before **(A)** and 5 min after **(B)** application of D-AP5 (200 µM). **(C, E)** Summarized data show spontaneous burst firing frequency **(C)** and spike number per burst **(D)** and membrane potentials **(E)** before (black) and after (red) application of D-AP5.

Next, we assessed the effect of D-AP5 on ongoing spontaneous burst firing. In these experiments, D-AP5 was applied after identifying neurons with spontaneous burst firing. D-AP5 did not significantly alter ongoing burst firing (**Figures 3A, B**). Burst firing frequencies (per min) were 18.68 ± 7.79 and 13.41 ± 4.10 at baseline and at least 3 min after D-AP5 application, respectively (n = 7, p = 0.28, paired t-test; **Figure 3C**). Spike number per burst was 7.93 ± 1.60 and 7.0 ± 2.20, respectively (p = 0.33, paired t-test; **Figure 3D**). The membrane potentials were not changed (baseline: -67.29 ± 2.45 mV; after D-AP5: -66.86 ± 2.10 mV; p = 0.76, paired t-test; **Figure 3E**).

### 3.3 NMDAR antagonists did not affect rebound burst firing

**Figure 4:**
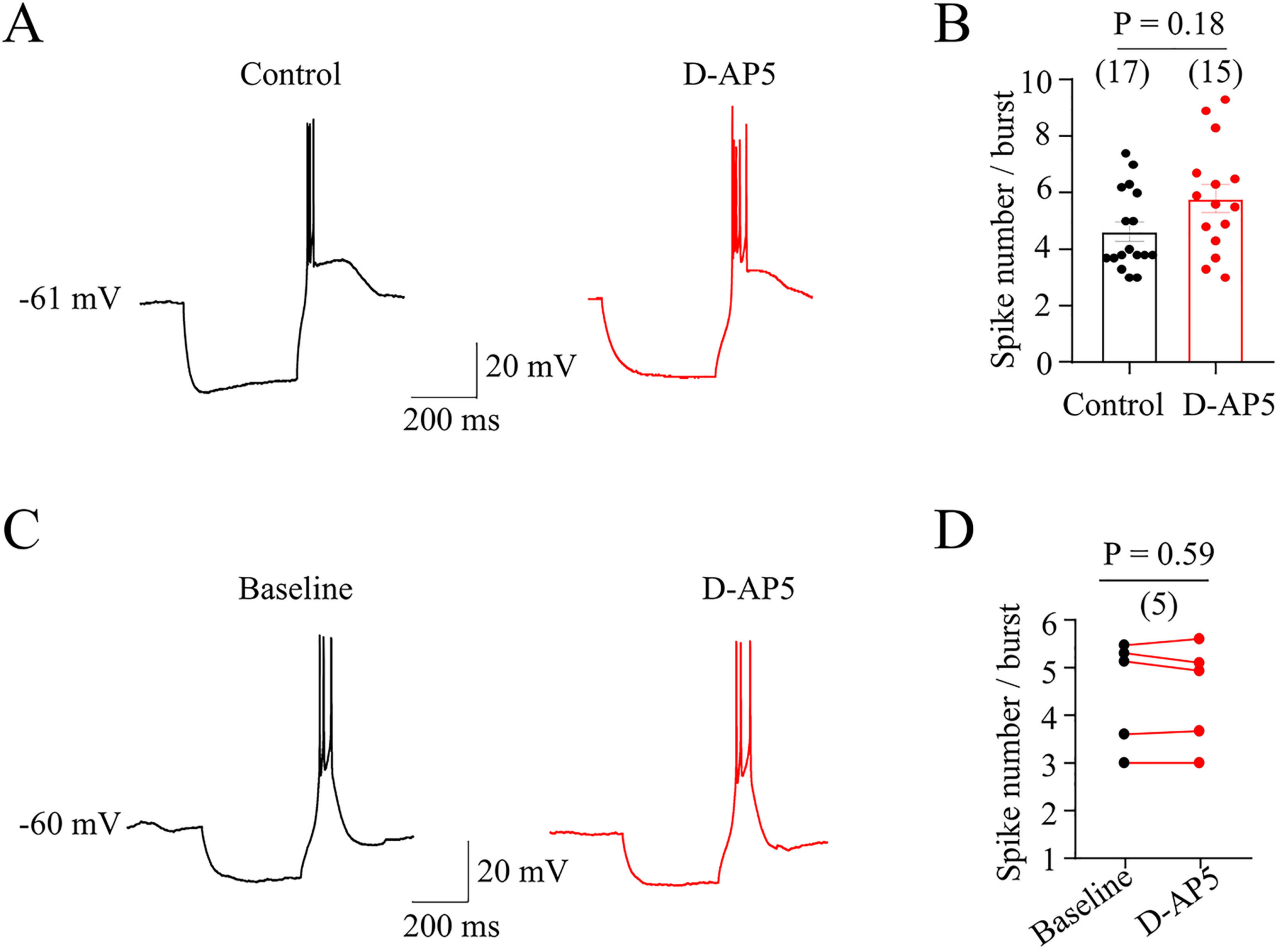
D-AP5 does not affect rebound burst firing in LHb neurons. **(A)** Representative rebound burst firing traces induced by hyperpolarizing pulses (350 ms, 60pA) in the absence (left, black) and presence (right, red) of D-AP5 in bath solution. **(B)** Summarized data show spike number per burst in the absence (black) and presence (red) of D-AP5. **(C)** Representative traces of rebound burst firing from a neuron before (left, black) and 5 min after (right, red) application of D-AP5. **(D)** Summarized data show spike number per burst before (black) and 5 min after (red) application of D-AP5.

Previous work showed that rebound burst firing following hyperpolarizing pulses induced depressive-like symptoms in naïve mice, and D-AP5 and ketamine disrupted rebound burst firing and alleviated depressive-like symptoms (5, 24), indicating that rebound burst firing also requires NMDAR activation and shares a similar role with spontaneous burst firing in depression. We recorded rebound burst firing by applying hyperpolarizing pulses (-40 to -100 pA, 350 ms) to neurons when holding the membrane potential between -60 mV and -75 mV. In the presence of D-AP5, rebound burst firing remained intact (**Figure 4A**). The percentage of neurons with rebound burst firing did not differ between the control (138/277) and D-AP5 groups (60/109; p = 0.36, Chi-square test). No significant difference was found in spike number per burst either (control: 4.6 ± 0.35, n = 17; D-AP5 group: 5.8 ± 0.50, n = 15; p = 0.18, K-S test; **Figure 4B**). We randomly sampled 17 neurons in control group and 15 neurons in D-AP5 group to compare spike number per burst. Application of D-AP5 did not affect spike number per burst during ongoing rebound burst firing (**Figures 4C, D**). Spike number per burst before and 3 min after application of D-AP5 was 4.5 ± 0.50 and 4.46 ± 0.48, respectively (n = 5, p = 0.59, paired t-test).

**Figure 5:**
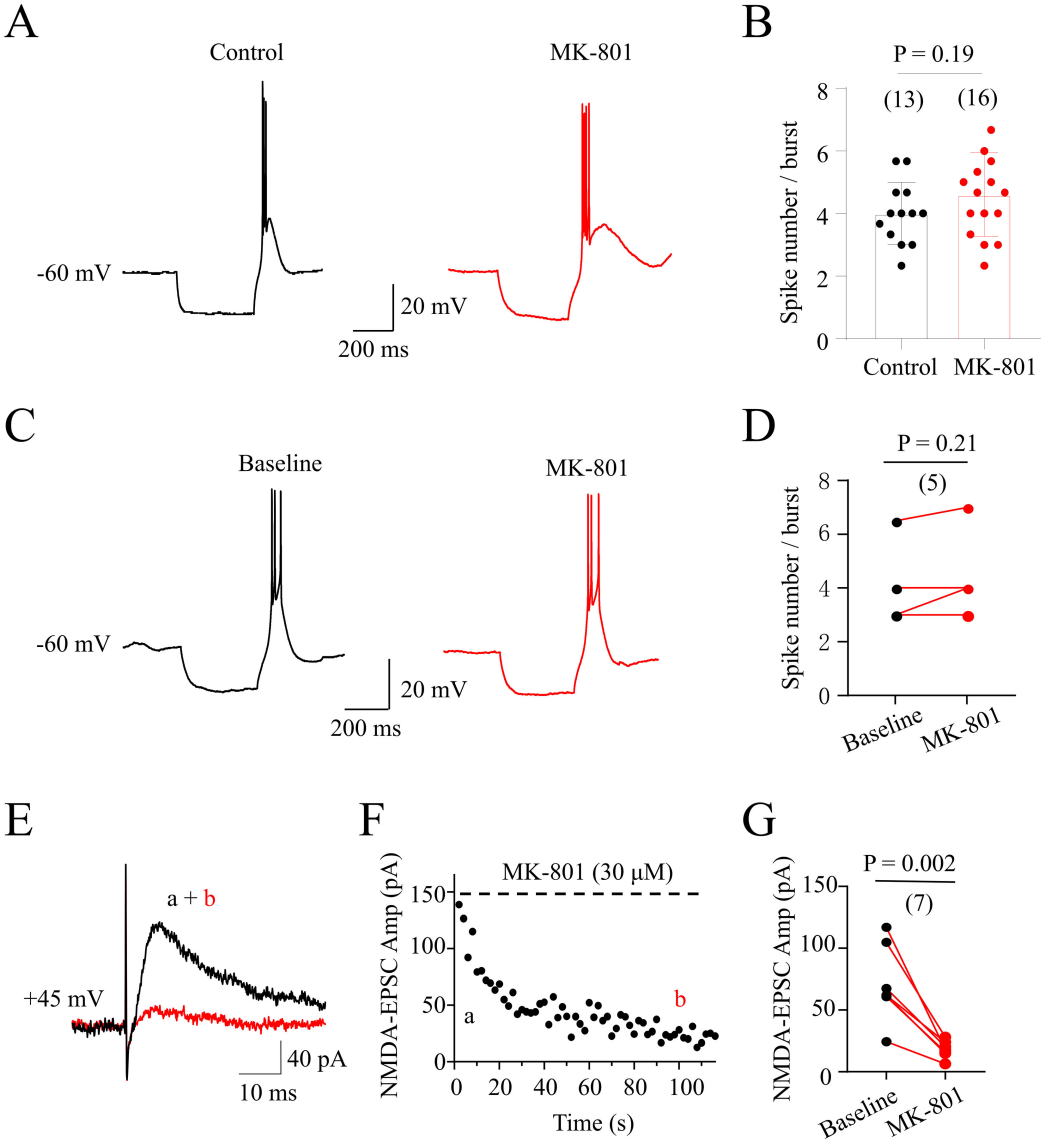
NMDAR antagonist MK-801 does not affect rebound burst firing of LHb neurons. **(A)** Representative rebound burst firing traces induced by hyperpolarizing pulses (350 ms, 60pA) in control (left, black) and MK-801(30µM) solution (right, red). **(B)** Summarized data show spike number per burst in control (black) and MK-801(red) groups. **(C)** Representative rebound burst firing traces recorded from a neuron before (left, black) and 5 min after (right, red) application of MK-801 (30µM). **(D)** Summarized data show spike number per burst for each neuron before (black) and after application of MK-801 (red). **(E)** Representative traces of NMDA component of evoked EPSC from a neuron before (black) and after (red) application of MK-801, taken at times marked by letters in **(F)**. **(F)** A representative experiment shows the time course of the effect of MK-801 on NMDA component of evoked EPSC. **(G)** Summarized data show NMDA component of evoked EPSC amplitude before (black) and at 2 min after (red) application of MK-801.  .

Like ketamine, MK-801 is a non-competitive NMDAR antagonist that acts in a use-dependent manner (25–27). Some previous studies also indicated that MK-801 has a rapid antidepressant effect (28, 29). We tested the effect of MK-801 on spontaneous and rebound burst firing in LHb neurons. We recorded 20 neurons in the presence of MK-801 (30 μM), 2 neurons exhibiting spontaneous burst firing, and 16 neurons with rebound burst firing. We made a comparison of spike number per burst between control and MK-801 groups, and found no significant difference (control: 4.0 ± 0.28, n = 13; MK-801: 4.6 ± 0.34, n = 16, p = 0.19, unpaired t-test; **Figures 5A, B**). Ongoing rebound burst firing induced by hyperpolarizing pulses was likewise unaffected (baseline: 3.9 ± 0.68; 5 min after MK-801: 4.2 ± 0.74; n = 5, p = 0.21, paired t-test; **Figures 5C, D**). To test the efficacy of MK-801, we recorded NMDA component of evoked EPSCs in the presence of 30 μM MK-801 in bath solution when neurons were held at +45mV. We averaged the first three NMDA components as the baseline. It was found that MK-801 significantly eliminated the amplitude of NMDA components evoked by 0.5 Hz stimulation in less than 2 min (baseline: 71.26 ± 11.65 pA; 2 min after application of MK-801: 18.20 ± 2.61 pA; n = 7, p = 0.002, paired t-test; **Figures 5E, G**).

## 4 Discussion

Previous studies have shown that both spontaneous and rebound burst firings in LHb neurons require the activation of NMDARs, and that blockade of NMDARs by ketamine or D-AP5 abolishes both forms of burst firing and exerts antidepressant effects in rodent models of depression (5, 6, 13). In contrast, our results show that neither spontaneous nor rebound burst firing in LHb neurons was blocked by NMDAR blockade. Specifically, D-AP5 failed to abolish burst firing when applied either before or during recording. We also examined the effect of MK-801 on rebound burst firing of LHb neurons and found that MK-801 did not affect rebound burst firing. Collectively, these results indicate that NMDAR activity is not necessary for the generation of burst firing in LHb neurons.

Our results contrast with earlier studies reporting that NMDAR antagonists, including D-AP5 and ketamine, completely abolished spontaneous burst firing in LHb neurons (5, 6, 13). Several factors may contribute to this discrepancy. First, differences in stress paradigms may be a contributor. We adopted a short-term restraint stress protocol, which reportedly does not induce depressive behaviors in mice (30), whereas previous studies employed a longer stress protocol that produces depressive phenotypes (30). It would be possible that NMDAR-dependent burst firing emerges preferentially under pathological conditions associated with depression. In support of this possibility, extrasynaptic NMDAR expression has been reported to be upregulated in LHb neurons in stress-susceptible or depressed animals, and the antidepressant effects of NMDAR antagonists appear restricted to depressed animals (28, 31). Second, membrane potential differences may influence the contribution of NMDARs to burst firing. In our experiments, spontaneous burst firing was frequently observed at hyperpolarized membrane potentials, conditions that strongly favor the activation of T-type calcium channels (23). Calcium spikes generated under these conditions may be sufficient to depolarize neurons to action potential threshold independently of NMDAR activation. Third, in previous studies (5), NMDAR antagonists were only perfused during burst firing recording, without examining burst firing under sustained NMDAR blockade. In our experiments, we recorded neurons in D-AP5 pre-treated slices, and in such way we could likely avoid some confounding variables affecting recording stability and burst firing, such as washout effect in a long whole-cell recording (32).

Previous studies showed that rebound burst firing of LHb neurons induced by optogenetic hyperpolarizing pulses led to depressive-like behaviors in naïve mice and was completely blocked by D-AP5 and Ketamine (5, 13). In contrast, we observed that rebound burst firing persisted in the presence of both D-AP5 and MK-801. In previous studies, rebound burst firing was recorded in naïve mice (5); thus, differences in animal state alone do not fully account for this discrepancy. At present, the mechanisms underlying these divergent observations remain unclear and warrant further investigation.

Burst firing is a widespread neuronal firing mode observed in multiple brain regions, including the cortex, hippocampus, thalamus, medial habenula, and midbrain dopaminergic nuclei (33). The ionic mechanisms underlying burst firing vary across regions: in cortical pyramidal neurons, bursts are largely mediated by NMDAR-dependent calcium spikes (20) whereas in medial habenula neurons, burst firing depends primarily on T-type calcium channels and is insensitive to NMDAR blockade (22). Our findings also suggest that T-type calcium channels play a primary role in rebound burst firing in LHb neurons.

Stress may dynamically regulate both T-type calcium channels and NMDARs. Chronic stress has been shown to enhance T-type calcium channel expression and burst firing in the ventral subiculum (34, 35), while early-life stress alters NMDAR activity in the LHb (31). Previous studies showed that T-type calcium channel expression in LHb was not changed in depressive animals, but RMP was hyperpolarized, which can enhance T-type calcium channel activation and burst firing (5, 6). Elucidating how different stress regimens reshape RMP, T-type calcium channel activity, and NMDAR function will help reconcile conflicting mechanistic conclusions from different laboratories.

Some limitations of the present study need to be acknowledged. First, we applied short-term restraint stress, rather than the chronic stress protocols used in landmark previous studies (5, 6); thus, the present results and cross-study comparisons must be interpreted with caution. Second, ketamine was not included in our experiments, leaving the direct effect of ketamine on LHb burst firing unaddressed. These unresolved issues warrant careful investigation in future follow-up studies.

In summary, our study demonstrates that blockade of NMDARs does not prevent or disrupt spontaneous or rebound burst firing in LHb neurons, indicating that NMDAR activity is not an essential prerequisite for the generation of burst firing in LHb neurons. These results contrast with previous reports and suggest that the contribution of NMDARs to burst firing in LHb may be context dependent. Further studies will be necessary to determine how neuronal state and stress history influence the mechanisms of burst firing and their relevance to depression.

## Data Availability

The original contributions presented in the study are included in the article/supplementary material. Further inquiries can be directed to the corresponding author.
